# Research on capital allocation efficiencies with four-dimensional factor capitals from China’s intelligent manufacturing enterprises

**DOI:** 10.1371/journal.pone.0270588

**Published:** 2022-07-21

**Authors:** Qiong Wang, Chengxuan Geng, Hai-tao E., Jiarui Song

**Affiliations:** 1 Aliyun School of Big Data, Changzhou University, Changzhou, Jiangsu, China; 2 School of Economics and Management, Nanjing University of Aeronautics and Astronautics, Nanjing, Jiangsu, China; University of Almeria, SPAIN

## Abstract

Compared with traditional manufacturing enterprises, intelligent manufacturing enterprises pay more attention to the investment of knowledge capital and technological capital. Taking 258 intelligent manufacturing listed companies in China from 2015 to 2020 as research samples, the paper selects the material capital, human capital, knowledge capital and technological capital of enterprises as the input variables of Cobb-Douglas production function. Considering that enterprises are often affected by spatial correlation, stochastic frontier panel model, spatial lag stochastic frontier panel model and dynamic spatial lag stochastic frontier panel model are constructed to measure capital allocation efficiencies of enterprises. The results show that all the factor capitals in the three models have a significant positive impact on enterprises’ performance, and the dual lag effect of time and space is significant. Moreover, it is more reasonable to use the dynamic spatial lag stochastic frontier panel model to estimate the parameters and measure capital allocation efficiencies. The development of intelligent manufacturing industry has significant space-time spillover effect among provinces. About 52.98% of intelligent manufacturing enterprises have high capital allocation efficiencies, but 12.04% still need to further optimize capital allocation. The gap between the actual performance of the sample enterprises and efficiency frontier is mainly due to technical ineffectiveness. From a regional perspective, the top ten enterprises with high capital allocation efficiencies are all in the eastern region, but the average of capital allocation efficiency is the highest in the western region, followed by the eastern and central regions.

## 1. Introduction

Under the wave of the fourth industrial revolution with rapid technological innovation, to seize the new development commanding height of the manufacturing industry, countries have formulated strategic plans to support and promote the development of intelligent manufacturing and reshape the global competitiveness of the manufacturing industry. For example, the National Scientific Research Project of intelligent manufacturing in the United States, the Horizon 2020 Plan of the European Union, and the Manufacturing 2050 Strategy of the UK take intelligent manufacturing in an important strategic direction. To accelerate the transformation and upgrading of the manufacturing industry, China put forward the strategic deployment of "Made in China 2025" in 2015, defining that intelligent manufacturing is the main direction to achieve a highly flexible production mode [1]. The strong strategic support from the government and the breakthrough of core technology have greatly reduced the application threshold of intelligent technology and promoted the vigorous development of the intelligent manufacturing industry. According to the survey data from the prospective industry research institute, the output value of China’s intelligent manufacturing industry has increased year by year, from less than 1 trillion yuan in 2015 to about 2.5 trillion yuan in 2020, with an average annual growth of about 25.25%.

With the rapid development of the intelligent manufacturing industry, there are also some problems. For example, intelligent manufacturing is still in the early stage [2]. And Li et al. (2019) regarded that the development of the intelligent manufacturing industry needs to absorb and train talents to build core competitiveness and ensure the intelligent process from the level of knowledge and technology [3]. Traditional manufacturing enterprises turn from scale expansion to technological innovation drive to improve their productivity, and emerging factor capital such as technological capital is the driving force for enterprises to carry out innovation activities, especially for the intelligent manufacturing industry. Employee knowledge, technical level, and management ability are becoming more and more important in intelligent manufacturing [4]. At present, while increasing the investment in intelligent manufacturing technology, enterprises have also begun to increase the investment in employee training and enterprise capability [5], showing a trend of paying more attention to emerging factor capital such as knowledge capital and technological capital. It can be seen that the intelligent manufacturing industry should not only pay attention to the coordinated development of the upstream and downstream of the industrial chain but also strengthen the organic integration and collaborative innovation of traditional factor capital and emerging factor capital, to promote the transformation and upgrading of industrial structure. The existing literature regards that the imbalance of capital allocation will lead to the decline of enterprise capital allocation efficiency, which is the main reason to hinder the transformation of manufacturing enterprises and the upgrading of manufacturing structure [6, 7]. Therefore, a correct understanding of the important role of emerging factor capital in intelligent manufacturing enterprises and optimizing the allocation of traditional factor capital and emerging factor capital will not only help to improve the efficiency of capital allocation but also promote the transformation of industrial structure and sustainable development.

In the existing literature, the research on capital allocation efficiency of manufacturing enterprises mostly adopts the Wurgler model [8], the production function method [9], and the idea of equalization of the marginal output of capital [10]. Few scholars combine the spatial econometric model with the stochastic frontier model to research the spatial measurement of enterprise capital allocation efficiency. New economic geography holds that almost all economic and geographical behaviors are spatially related, especially when the two regions are close in geographical location or similar in economic characteristics. If we ignore the spatial relationship of capital allocation and take each region as an independent sample to investigate the capital allocation efficiency of intelligent manufacturing enterprises, it may not accurately reflect the real efficiencies. In addition, most literature takes labor input and capital input as input factors and does not include emerging factor capitals such as knowledge capital and technological capital in the production function. Therefore, it is necessary to introduce the spatial correlation effect of capital allocation activities, take traditional factor capital and emerging factor capital as inputs, measure the capital allocation efficiency of Chinese intelligent manufacturing enterprises more objectively and reasonably, and further analyze the impact of different factor capital on enterprise performance, which is of great significance to optimize the capital allocation of intelligent manufacturing industry, improve capital allocation efficiencies, and promote the transformation and upgrading of the manufacturing industry.

The rest of this paper is organized as follows. In the next section, the literature review focuses on the relevant research on multi-factor capitals and capital allocation efficiencies of enterprises. In the following section, the relevant variables, methods, and samples are provided. In the third part, we discuss the results of empirical research. Finally, it is our conclusions.

## 2 Literature review

### 2.1 Enterprise factor capitals

With the development of western economic theory, the role of capital in economic growth has experienced three stages: "material capital as the main body", "human capital" as the center, and "knowledge capital as the core". According to the theory of factor capital, traditional factor capital includes human and material capital, while technological and knowledge capital are called emerging factor capitals. With the development of western economic theory and the information age, emerging factor capitals are separated from traditional factor capitals and gradually occupy a dominant position. The research on enterprise factor capitals can be summarized into two categories.

Scholars have done much research on factor capitals at the enterprise level. Schultz (1960) first expounded on the concept of human capital and regarded that human capital referred to people with knowledge and work skills [11]. The contribution of human capital is more important compared with the amount of material capital and labor force. Barro & Lee (1993) proposed that human capital can be formed through education, training, and learning by doing [12]. At present, the measurement methods of human capital include the education stock method, retrospective cost method, and expected income measurement method. The education stock method refers to measuring the human capital of the labor force by education level or years [13]. Naro et al. (2019) regarded that the degree of education can reflect people’s labor ability [14]. The retroactive cost method is a method to measure the cost incurred in the formation of human capital under the principle of historical cost [15, 16]. The expected income measurement method refers to measuring the human capital of the labor force through the total wages that the labor force will receive for the services provided in the future [17]. Liu & Huang (2018) measured enterprise human capital by the cash paid to and for employees [18].

Knowledge capital was first proposed in 1956 by Galbraith, an American economist [19]. Stewart (1998) concludes that intellectual capital is knowledge, information, intellectual property, and experience to create wealth [20]. The measurement of knowledge capital includes the measurement based on input and expenditure indicators [21] and based on innovation achievement indicators [22, 23]. Yu and Wang (2020) observed that compared with human capital, knowledge capital investment would bring enterprises more uncertainty about the change in technological innovation efficiency [24]. The measurement research of technological capital is mainly based on R&D expenditure and technological capitalization [25–27].

Scholars have conducted a lot of research on the impact of different factor capitals on enterprises and obtained different conclusions. Among them, there are many relevant studies on the impact of material capital and human capital on enterprise performance [28, 29]. Miller & Upadhyay (2000) noticed that human capital can promote the improvement of a country’s total factor productivity significantly [30]. Li et al. (2019) took the university enrollment expansion policy as a policy variable and found that the increase of human capital will promote the R&D investment of enterprises, improve the technical level of enterprises, and then drive the improvement of total factor productivity [31].

With the continuous emergence of the role of knowledge capital in enterprise development, more and more scholars begin to pay attention to its relationship with enterprise performance. Griliches (1984) measured knowledge capital by R&D capital and proposed the endogenous growth theory with knowledge capital and innovation as the engine of enterprise growth [32]. Compared with physical capital, the return on investment of enterprise R&D capital is higher, and the spillover effect of social return is also higher [33]. Some scholars also obtained that knowledge capital can promote enterprise growth effectively [34].

The theoretical research of technological capital mostly focuses on the construction of its connotation system [35]. And the empirical research of technological capital mainly focuses on its measurement and action mechanism. Based on the Solow model, Ellen & Edward (2009), and Xu (2017) discussed the contribution of technological capital to economic growth and interpreted technological capital as one of the driving forces of enterprise value [25, 36]. Technological capital is significantly positively correlated with enterprise growth and value. Wang & Qi (2016) observed that only invention patents with a high degree of innovation had a significant positive effect [37]. Human capital and technological capital have a strong linkage. Wang & Luo (2017) regarded that high-quality human capital can promote technological innovation and the R&D performance of enterprises and provide more value for enterprise innovation [34]. Luckstead (2014) noticed that technological capital and human capital played an important role in US productivity growth by adjusting the investment in material capital through technical factors [38].

### 2.2 Enterprise capital allocation efficiency

For capital allocation efficiency, many scholars discussed and made some expansions to two kinds of methods including the marginal output equilibrium method [39] and the input-output method [40, 41]. For example, Galindo (2007) measured the efficiency of enterprise capital allocation through the sales revenue or profit obtained by unit capital and concluded that the financial freedom of most developing countries significantly promoted the efficiency of enterprise capital allocation [42]. Fan et al. (2017) measured the inter-provincial capital allocation efficiency through the marginal capital-output ratio and concluded that there was a nonlinear relationship between international technological spillovers and capital allocation efficiencies [43]. Yin et al. (2021) regarded that executive compensation played an inverted U-shaped role in resource allocation efficiencies of enterprises [44]. Cheng, et al. (2020) employed the residual of the regression, the difference between actual investment and expected investment to measure the efficiency of enterprise capital allocation, and considered that the compensation incentive of management at different life cycle stages has different effects [45]. Li & Wang (2020) calculated the factor allocation efficiency of China’s service industry according to the stochastic frontier model, taking capital factors, labor factors, and energy factors as inputs, and considered that the capital factor allocation efficiency promoted the factor allocation efficiency of the service industry, while the labor factor allocation efficiency inhibited [46].

Throughout the existing literature, some research results have been obtained in the aspects of enterprise factor capital and allocation efficiency. It has laid a good foundation for this paper. For the capital allocation efficiencies of enterprises, although many scholars have mentioned paying more attention to improving enterprise knowledge capital, R&D investment, and technological innovation [34, 47]. However, little research on capital allocation efficiency takes emerging factor capital as input. The existing research lacks quantitative research on the organic integration of enterprise factor capital combined with the spatial econometric model. Therefore, based on the related research, this paper constructed a spatial stochastic frontier panel model with the traditional factor capital and emerging factor capital of listed enterprises as inputs to investigate the spatial measurement of capital allocation efficiency of China’s intelligent manufacturing enterprises.

## 3. Data and methodology

### 3.1 Data source and sample selection

This paper selected listed enterprises belonging to the concept of intelligence in all A-share manufacturing industries in Shanghai and Shenzhen from 2015 to 2020 as the initial sample. To ensure the continuity and effectiveness of data, the initial samples are screened according to the following rules. (1) Exclude the enterprises with incomplete data disclosure and zero data. (2) Eliminate ST-listed enterprises. (3) Winsorize the main continuous variables tailed at a 1% level to eliminate the influence of variable outliers on the research conclusions. Finally, 258 intelligent manufacturing listed enterprises are selected. There are 1548 groups of effective research data. The enterprise financial data in the paper came from the CSMAR database, the Wind database, and the annual financial reports of listed enterprises.

### 3.2 Variable design

#### 3.2.1 Dependent variable

Take enterprise performance as the dependent variable. From previous relevant literature [48, 49], enterprise performance is measured by operating income to reflect the business scale and operation status of intelligent manufacturing enterprises in this paper.

#### 3.2.2 Independent variables

Material capital (PC). Material capital is the factor capital in the form of production materials. This paper chose the sum of the book value of fixed assets, inventory, and investment real estate to measure the material capital.

Human capital (HC). Human capital is one of the important capitals for the long-term sustainable development of enterprises. It reflects the capital elements owned by enterprises that can increase the value of enterprises. The related literature mainly measured human capital through the educational background, working years, and employee compensation of enterprise executives. In this paper, human capital refers to the value of the human capital of core employees, the value invested in recruiting and training core employees, and the transfer value paid to retain senior executives and core technicians. Considering the characteristics of the intelligent manufacturing industry and relevant literature [36, 50], this paper employed the total annual remuneration paid by the enterprise to directors, supervisors, senior managers, and core technicians. It includes the effect of knowledge accumulation.

Knowledge capital (KC). Knowledge capital is the sum of the value of knowledge capital owned by an enterprise, which is composed of the new value created by labor in the process of R&D, the transfer value of monetary capital, the new value of absorbing relevant knowledge, and the remaining stock of intangible assets deducting technological capital. Cheng & Lu (2014) measured the knowledge capital investment of listed companies through relevant technological development and transferring funds [51]. Xu (2017) measured the net value of relevant intangible assets. Technology transfer is the most common way of knowledge flow [36]. This paper regards that enterprise knowledge capital also includes technology introduction funds, technology transformation funds, technology purchase funds at home and abroad, as well as digestion and absorption funds. Therefore, the sum of the net book value of intangible assets, technology purchase funds, and technology introduction funds is selected to measure knowledge capital.

Technological capital (TC). Due to the strong dependence on technology and the complexity of technology expression, the measurement indicators of technological capital in relevant literature are not consistent. Ellen & Edward (2009) used the sum of patent, nonpatent technology, trademark, and other technical forms as the investment of enterprise technological capital [25]. At that time, the trademark has not been widely adopted. Luo (2014) viewed that trademarks reflected the concept of enterprises and should belong to knowledge capital, and included the development expenditure of systems and software into technological capital [52]. Technological capital is an important part of improving the core competitiveness of enterprises, with diversified forms of expression. Enterprises generally accumulate technology through independent research and development. Therefore, this paper employed R&D funds to measure technological capital. Specific variables are shown in Table 1.

**Table 1:** Definition and formula of variables (unit: yuan).

Variable type	Variable name	Symbol	Formula
**Dependent variable**	Enterprise performance	Y	Operating revenue
**Independent variable**	Material capital	PC	The sum of the net value of fixed assets, inventory, and the book value of investment real estate
	Human capital	HC	The total annual remuneration of directors, supervisors, senior managers, and technicians
	Knowledge capital	KC	The sum of the net book value of intangible assets, technological introduction funds, technological transferring funds, and technological purchase funds, as well as digestion and absorption funds
	Technological capital	TC	Total annual R&D expenditure of the enterprise

### 3.3 Empirical model

The stochastic frontier method developed by Battese & Coelli (1995) [53] is the most widely used in practice. In most existing literature, the Cobb-Douglas production function is employed as the basic form of the stochastic frontier production function. To investigate capital allocation efficiencies of intelligent manufacturing enterprises in China, the paper introduces four input factors: material capital, human capital, knowledge capital, and technological capital into the stochastic frontier production function, and constructs a stochastic frontier panel model as the following:

$$\ln y_{it}=\beta_{1}\ln PC_{it}+\beta_{2}\ln HC_{it}+\beta_{3}\ln KC_{it}+\beta_{4}\ln TC_{it}+v_{it}-u_{it}$$ (1)

In Model 1, the dependent variable *y*_*it*_ represents the total operating revenue and reflects the output of the *i*th intelligent manufacturing enterprise in period *t* (*i* = 1,2,…,*N*;*t* = 1,2…,*T*). *N* denotes the number of enterprises. And *T* is the number of years. The explanatory variable PC represents the material capital of the enterprise, which is measured by the sum of the net value of fixed assets, inventory, and the book value of investment real estate, and reflects the fixed assets, material materials, and other elements invested by the enterprise. HC refers to the heterogeneous human capital of the enterprise, which is measured by the sum of the remuneration of directors, supervisors, and senior executives. KC refers to enterprise knowledge capital, which is measured by the sum of R&D funds and technology digestion and absorption funds. TC refers to the technological capital of an enterprise, which reflects the comprehensive technical level of the enterprise by measuring the net book value of intangible assets, technology purchase funds, and technology introduction funds. *β* is the model parameter to be estimated, reflecting the output elasticity of the corresponding factor capital. The disturbance term in the model is a mixture of an inefficiency term and idiosyncratic error. Intelligent manufacturing enterprises combine their heterogeneity characteristics to continuously improve their efficiency level, so it is necessary to introduce a function to determine how the ineffective effect changes with time. Here we use the functions of Battese & Coelli (1992) [54]. *v*_*it*_ is the stochastic disturbance term, reflecting the error caused by uncontrollable external factors and following the Normal distribution $N(0,\sigma_{v}^{2})$. The parameter $u_{it}=u_{i}e^{-\eta (t-T)}$ is the technical inefficiency term, reflecting the degree of inefficiency of enterprise capital allocation, which *u*_*i*_ follows a truncated normal distribution $N^{+}(\mu ,\sigma_{u}^{2})$. The capital allocation efficiency of Enterprise *i* in the *t* year is expressed as $Eff_{it}=e^{-u_{it}}$. And $\gamma =\sigma_{u}^{2}/(\sigma_{u}^{2}+\sigma_{v}^{2})\in [0,1]$ reflects the proportion of technical inefficiencies in the technical inefficiency term. When the estimated value tends to 1, it shows that the gap between the actual performance and the frontier of the enterprise mainly comes from the loss caused by technical inefficiency. On the contrary, it is mainly due to statistical error. Therefore, the value *γ* can test whether the model selection is reasonable.

The intelligent manufacturing industry is mostly supported by technological breakthroughs and development. Spatial technological spillovers often occur in the process of technology promotion. Therefore, the capital allocation efficiency of intelligent manufacturing enterprises is affected not only by their factor capital but also by the spatial spillover of geographical proximity regions. It causes the traditional panel data model may have serious setting errors and cannot effectively capture the main capital variables affecting the capital allocation of intelligent manufacturing enterprises. Therefore, to better measure the capital allocation efficiencies of intelligent manufacturing enterprises, the spatial lag term of the explained variable is introduced to construct the spatial lag stochastic frontier panel model:

$$\ln y_{it}=\rho W\ln y_{it}+\beta_{1}\ln PC_{it}+\beta_{2}\ln HC_{it}+\beta_{3}\ln KC_{it}+\beta_{4}\ln TC_{it}+v_{it}-u_{it}$$ (2)

In Model 2, *ρ* is the spatial autoregressive coefficient of the explained variable. *v*_*it*_ is a stochastic disturbance term, reflecting the error caused by uncontrollable external factors and following the Normal distribution $N(0,\sigma_{v}^{2})$. *u*_*it*_ is the technical inefficiency item, reflecting the degree of inefficiency of enterprise capital allocation, which *u*_*i*_ follows a truncated normal distribution $N^{+}(\mu ,\sigma_{u}^{2})$. *W* is the *N*-order spatial weight matrix, reflecting the spatial correlation between different enterprises. In this paper, the bisection adjacent rule is applied to define the spatial weight matrix *W*. If the provinces where two enterprises are located are adjacent, the corresponding matrix element is assigned as 1, otherwise, it is assigned as 0. In addition, the element on the main diagonal in the spatial weight matrix is also assigned as 0. *W* ln *y*_*it*_ is the spatial lag dependent variable.

The technological upgrading process of the intelligent manufacturing industry often has time continuity. And there is a time delay in the whole process from technological R&D to achievement transformation to acceptance by the market. Therefore, with different times and spaces, the values of various factor capitals of enterprises are also different. Based on model 2, the time-space lag term of the explained variable is introduced to construct the dynamic spatial lag stochastic frontier panel model:

$$\ln y_{it}=\rho W\ln y_{it}+\lambda W\ln y_{i,t-1}+\beta_{1}\ln PC_{it}+\beta_{2}\ln HC_{it}+\beta_{3}\ln KC_{it}+\beta_{4}\ln TC_{it}+v_{it}-u_{it}$$ (3)

In Model 3, *W* ln *y*_*it*_ is the spatial lag dependent variable and *ρ* is the spatial autoregressive coefficient. *W* ln *y*_*t*−1_ is the time-space lag term of the dependent variable and *λ* is the time-space lag coefficient of the explained variable. Referring to the relevant literature [55], the parameters in the above models are estimated by maximum likelihood estimation.

## 4 Results and analysis

### 4.1 Descriptive statistics

To compare the knowledge capital and technology capital among different enterprises, Table 2 gives the distribution of the proportion of knowledge capital and technology capital to operating revenue in intelligent manufacturing enterprises. The proportion of knowledge capital and technology capital varies greatly among different intelligent manufacturing enterprises. The proportion of knowledge capital of 1053 sample enterprises is between 0 and 10%, accounting for 68.02% of the total sample. The proportion of technological capital of 1339 sample enterprises is distributed between 0–10%, accounting for 86.50% of the total sample. Therefore, all the variables are processed by taking the natural logarithm in the model. In this way, it conforms to the form of Douglas production function. Moreover, the nature and correlation of sample data will be kept, and the collinearity and heteroscedasticity of variables in the model will be weakened to make the data more stable.

**Table 2:** Distribution of the proportion of knowledge capital and technology capital to operating revenue.

Ratio	KC/Y		TC/Y	
	Sample size	Sample ratio	Sample size	Sample ratio
**[0, 5%)**	552	35.66%	856	55.30%
**[5%, 10%)**	501	32.36%	483	31.20%
**[10%, 15%)**	201	12.98%	98	6.33%
**[15%, 20%)**	94	6.07%	40	2.58%
**[20%, 25%)**	52	3.36%	18	1.16%
**[25%, 30%)**	34	2.20%	13	0.84%
**[30%, 35%)**	25	1.61%	11	0.71%
**[35%, 40%)**	10	0.65%	1	0.06%
**[40%, 45%)**	13	0.84%	15	0.97%
**[45%, 50%)**	12	0.78%	1	0.06%
**[50%, +∞)**	55	3.49%	12	0.78%

According to the descriptive statistics in Table 3, the knowledge capital and technological capital of the intelligent manufacturing industry are higher than those of other industries. The average values of enterprise knowledge capital and technological capital are 683 million yuan and 503 million yuan respectively, and the standard deviations are 1.83 billion yuan and 1.39 billion yuan respectively. The maximum and minimum values are quite different, indicating that different intelligent manufacturing enterprises have significantly different knowledge capital and technological capital.

**Table 3:** Descriptive statistical results of variables (unit: yuan).

Variable	Obs	Mean	Std.	Min	Max
**Y**	1548	1.36e+10	5.56e+10	3.17e+07	8.88e+11
**PC**	1548	3.36e+09	1.20e+10	7624327	1.43e+11
**HC**	1548	7972770	8369153	429000	8.56e+07
**KC**	1548	6.83e+08	1.83e+09	2115195	1.62e+10
**TC**	1548	5.03e+08	1.39e+09	283305.9	1.59e+10

### 4.2 Empirical analysis

#### 4.2.1 Model estimation results

To avoid pseudo regression results in the panel data model, the unit root test is performed on all variables in the model. It showed that all variables in the model are stationary series after taking the natural logarithm.

The empirical results of the maximum likelihood estimation of Model 1 are given in column 2 of Table 4. From the results, the chi-square statistic of Model 1 is 48922.02, and the estimated value of the log-likelihood function is –753.1806, which are all significant at the 1% level. It indicates that the model has passed the test as a whole. All output elasticity coefficients of material capital, human capital, knowledge capital, and technological capital are significantly positive at the level of 1%. It indicates that the all factor capitals of intelligent manufacturing enterprises have a significant positive effect on their output. Among them, the output elasticity of human capital is the highest, which is 0.8063, followed by technological capital, material capital, and knowledge capital. The model parameter $\gamma =\sigma_{\mu }^{2}/(\sigma_{\mu }^{2}+\sigma_{v}^{2})$ is 0.9036, and the parameter $\sigma_{\mu }^{2}=0.7505$ is much greater than $\sigma_{v}^{2}=0.0801$. It shows that there is a significant technical inefficiency in sample enterprises. The technical inefficiency reflects the inefficiency of capital allocation efficiencies of enterprises. Although the stochastic disturbance term *v*_*it*_ and the technical inefficiency term *u*_*it*_ determine jointly the error term *ε*, the gap between the actual performance of enterprises and the efficient frontier mainly comes from the loss caused by its technical inefficiency. OLS estimation ignores the potential technical inefficiency, which shows that it is reasonable to use maximum likelihood estimation for Model 1.

**Table 4:** Estimation results of the models.

Variables	Model 1: Stochastic frontier panel model	Model 2: Spatial lag stochastic frontier panel model	Model 3: Dynamic spatial lag stochastic frontier panel model
**lnPC**	0.1543*** (0.0160)	0.1482*** (0.0159)	0.1476*** (0.0158)
**lnHC**	0.8063*** (0.0291)	0.7982*** (0.0289)	0.7969*** (0.0287)
**lnKC**	0.1330** (0.0241)	0.1391*** (0.0238)	0.1399*** (0.0237)
**lnTC**	0.1653*** (0.0234)	0.1559*** (0.0233)	0.1557*** (0.0229)
***ρ***	—	0.00005*** (0.00001)	–0.0261*** (0.0094)
***λ***	—	—	0.0263*** (0.0095)
***σ* ^2^**	0.8306*** (0.0719)	0.8403*** (0.0745)	0.8388*** (0.0746)
***γ***	0.9036*** (0.0091)	0.9063*** (0.0091)	0.9067*** (0.0090)
$\sigma_{u}^{2}$	0.7505*** (0.0719)	0.7616*** (0.0745)	0.7605*** (0.0746)
$\sigma_{v}^{2}$	0.0801*** (0.0032)	0.0787****** (0.0031)	0.0783*** (0.0031)
**Log- likelihood**	–753.1806***	–743.9456***	–740.2053***
**Wald chi2**	48922.02***	89430.75***	124396.82***

Note

* * *, * *, and * indicate significance at the level of 1%, 5%, and 10% respectively, and the standard error is in parentheses.

However, if there is a spatial effect in the sample data, the estimation of coefficients in Model 1 may be biased. Therefore, the Moran index is adopted to test whether there is a spatial correlation in the intelligent manufacturing industry. A global spatial autocorrelation test is performed on the explained variables, and the specific results are shown in Table 5. The test results of the Moran index show that all the p-value of the Moran index of the explained variables from 2015 to 2020 are less than 10%, and the Moran indexes are less than 0. It indicates that there is a significant spatial divergence effect and large spatial difference in the development of the intelligent manufacturing industry among provinces. Through the local spatial autocorrelation test, it is found that the Moran index of some intelligent manufacturing enterprises is significant at the level of 1%. This is consistent with the test result of global spatial autocorrelation. Therefore, it is necessary to introduce the spatial stochastic frontier panel model for research.

**Table 5:** Test results of spatial correlation of intelligent manufacturing enterprises.

Index	2015	2016	2017	2018	2019	2020
**Moran’s I**	–0.017*	–0.019**	–0.0187*	–0.0187*	–0.0197*	–0.0187*
**P-value**	0.066	0.040	0.062	0.067	0.072	0.082

Note

* * *, * *, * are significant at the level of 1%, 5% and 10% respectively.

Column 3 in Table 4 shows the empirical results of Model 2. After introducing spatial correlation, the chi-square statistic of Model 2 is 89430.75, and the estimated value of the logarithmic likelihood is –743.99456, both of which are significant at the 1% level. It indicates the model has passed the test as a whole. The estimated value of the log-likelihood function in Model 2 is better than that in Model 1. The output elasticity coefficient of each factor capital is significantly positive at the 1% level, indicating that the four-factor capitals of intelligent manufacturing enterprises have a significant positive effect on their performance. The spatial self-regression coefficient *ρ* is significantly positive at the 1% level, which is inconsistent with the results of the above Moran index test. From the value of parameters *γ*, $\sigma_{\mu }^{2}$, and $\sigma_{v}^{2}$, the gap between the actual performance and efficiency frontier of sample enterprises is still mainly due to technical inefficiency.

Column 4 in Table 4 shows the empirical results of Model 3. After introducing the dual lag effect of time and space, the chi-square statistic of Model 3 is 124396.82, and the estimated value of the log-likelihood function is −740.2053, which are all significantly indigenous at the 1% level. It indicates that the model has passed the test as a whole. The output elasticity coefficient of each factor capital in Model 3 is significantly positive at 1% level, indicating that each factor capital has a significant positive effect on enterprise performance. The output elasticity coefficient of human capital is 0.7969, significantly higher than other factor capitals, followed by the output elasticity coefficient of technological capital is 0.1557, material capital, and knowledge capital. Knowledge capital and technological capital have synergistic and balanced effects. The contribution of technological capital to intelligent manufacturing enterprises is greater than that of knowledge capital, but there is little difference between them. It is consistent with the findings of Wang & Luo (2017) [34], as well as consistent with the estimation results of Model 1 and Model 2. And it is also in line with the expected factor output. The spatial self-regression coefficient is −0.0261, and the time-space double lag coefficient is 0.0263, which are all significant at the 1% level. Moreover, it is consistent with the results of the Moran index test. It shows that the development of the intelligent manufacturing industry has a significant time-space spillover effect among provinces, which has a significant role in promoting the development of the intelligent manufacturing industry. The parameter $\sigma_{u}^{2}$ is much larger than $\sigma_{v}^{2}$, and the estimated value *γ* is about 0.90. It indicates that the intelligent manufacturing industry has obvious inefficiency in capital allocation, which is consistent with the estimation results of Model 1 and Model 2. The estimated value of Model 3 is greater than that of Model 1 and Model 2, indicating that Model 3 is more significant for inefficiencies. In addition, although the estimated values of the output elasticity of each factor capital in the three models are not significantly different, the estimated value of the log-likelihood function in Model 3 is better than the other two models. In summary, the estimation results of the output elasticity coefficient of each factor capital in Model 3 are more convincing.

#### 4.2.2 Distribution characteristics of capital allocation efficiency

**Fig 1:**
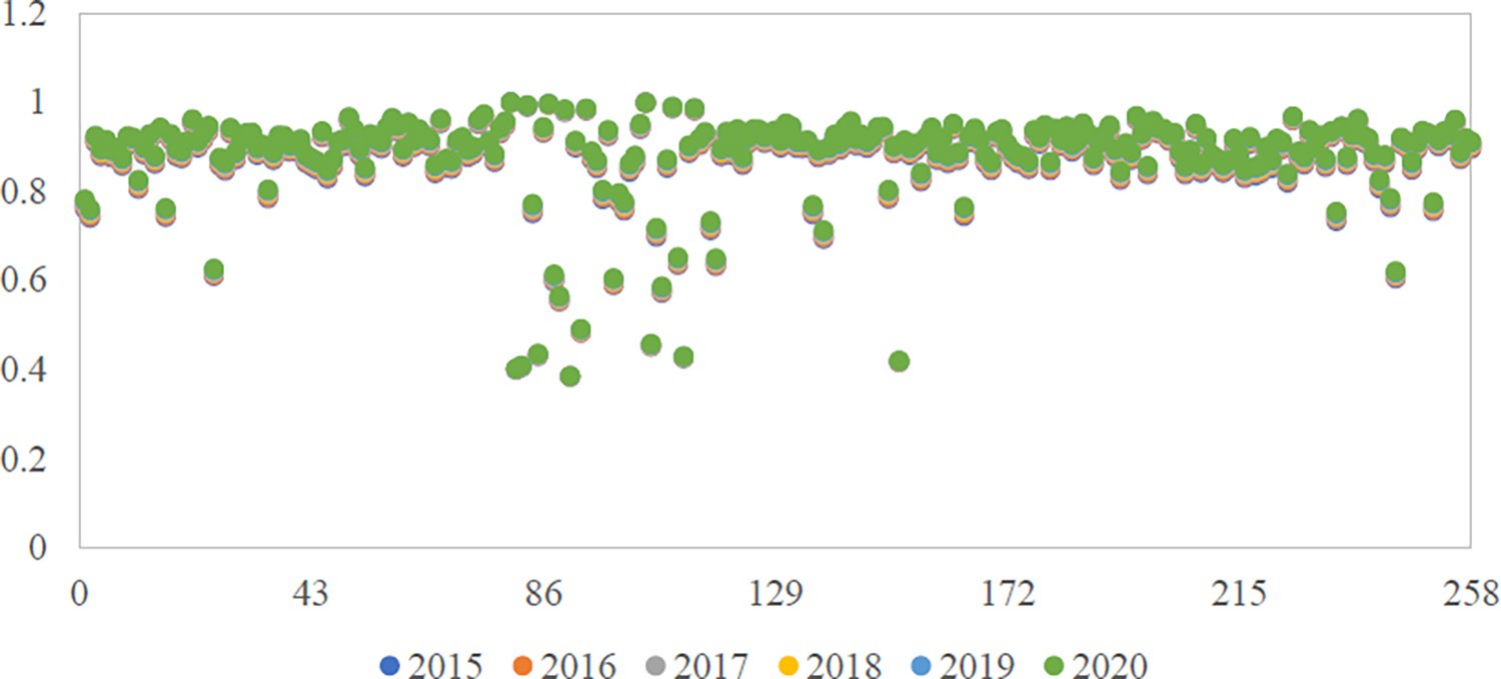
Distribution of capital allocation efficiency of enterprises from 2015 to 2020.

After comparing the above models, the dynamic panel spatial lag stochastic frontier model is selected to calculate the capital allocation efficiency of the intelligent manufacturing industry. Then the capital allocation efficiency values of 258 intelligent manufacturing enterprises from 2015 to 2020 are obtained. See Fig 1 for details.

From Fig 1, the efficiency of all capital allocation of intelligent manufacturing enterprises from 2015 to 2020 is less than 1, indicating that the actual output of intelligent manufacturing enterprises has not reached the most effective output level, and the factor capital allocation needs to be further optimized. On the whole, the estimated capital allocation efficiencies of enterprises are between 0.3840 and 0.9993, and the overall average value is 0.8740, which is at the upper-middle level. About 52.98% of enterprises’ capital allocation efficiency is greater than or equal to 0.9, and about 12.04% of enterprises’ capital allocation efficiency is less than 0.8. From a regional perspective, enterprises with the top ten capital allocation efficiencies are located in the eastern region, but the average capital allocation efficiency of intelligent manufacturing enterprises in the western region is the highest, followed by the eastern and central regions.

**Fig 2:**
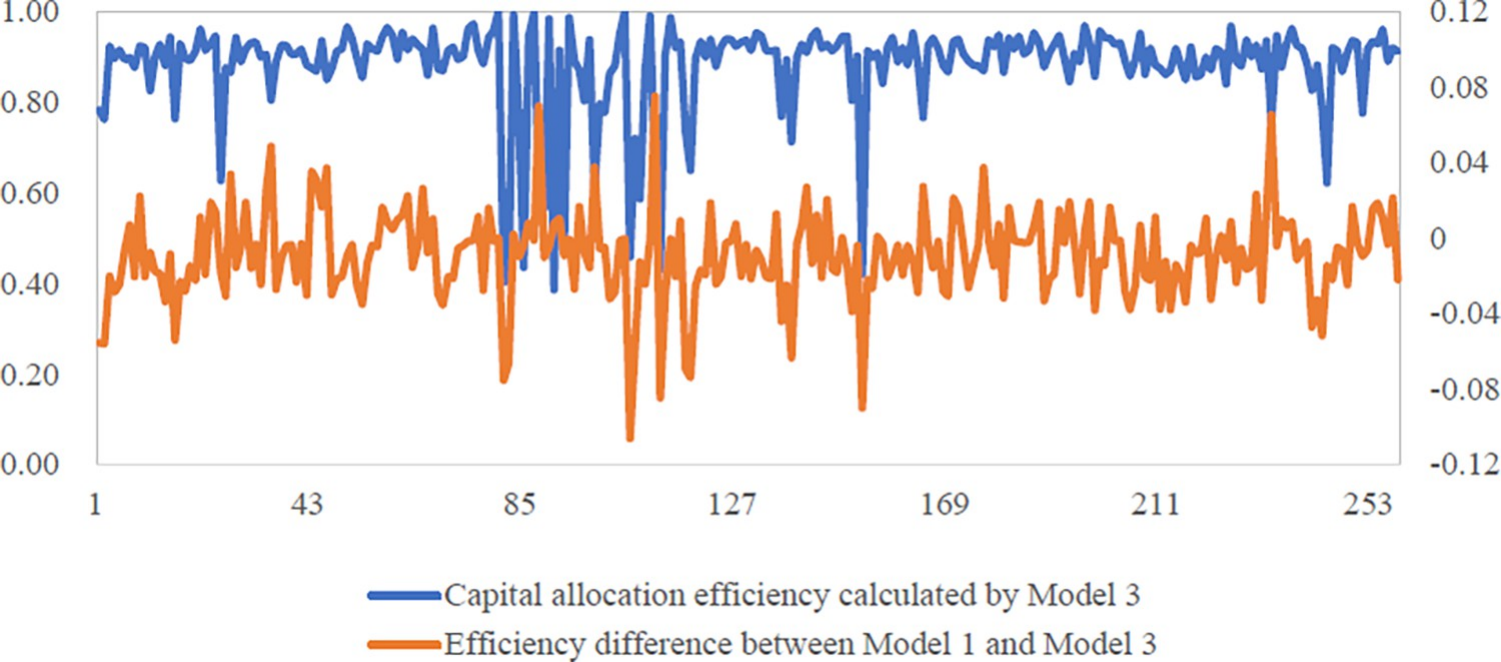
Distribution of differences in capital allocation efficiency between Model 1 and Model 3.

Fig 2 shows the distribution of difference with capital allocation efficiencies of intelligent manufacturing enterprises in 2020 using Model 1 and Model 3. It can be easily observed from Fig 2 that after introducing the time-space double lag term, the fluctuation range of the estimated capital allocation efficiencies is slightly reduced and tends to be assimilated. Both the spatial autoregressive coefficient *ρ* and the time-space lag coefficient *λ* in Model 3 show significant spatial correlation. Therefore, the differentiation of capital allocation efficiency estimates may be due to the bias caused by ignoring the spatial effect in the panel stochastic frontier model. Compared with the results of Model 1, capital allocation efficiencies of about 74.21% intelligent manufacturing enterprises decreases, but not significantly.

### 4.3 Robust test

To ensure the robustness of the research conclusion, the following robustness tests are carried out in this paper. Using enterprise value instead of total operating income as the measurement index of enterprise output, and using the total wages paid by the enterprise in each year as the measurement index of human capital, it is substituted into the above three models for parameter reestimation. The specific results are shown in Table 6. The results show that the regression coefficients of the four-factor capital are significantly positive. The output elasticity of human capital is the largest, followed by technological capital and knowledge capital. Moreover, the spatial autoregressive coefficient and time-spatial lag coefficient in Model 3 are significant. It is not substantially different from the previous conclusion. Therefore, it can be considered that the main conclusions are robust.

**Table 6:** Estimated results for robustness test.

Variables	Model 1: Stochastic frontier panel model	Model 2: Spatial lag stochastic frontier panel model	Model 3: Dynamic spatial lag stochastic frontier panel model
**lnPC**	0.1687*** (0.0288)	0.1686*** (0.0288)	0.1682*** (0.0287)
**lnHC1**	0.5097*** (0.0394)	0.5110*** (0.0393)	0.5131*** (0.0394)
**lnKC**	0.1960*** (0.0354)	0.1972*** (0.0354)	0.1954*** (0.0354)
**lnTC**	0.4226** (0.0376)	0.4158*** (0.0378)	0.4162*** (0.0378)
***ρ***	—	0.000008* (0.000004)	–0.00110* (0.0008)
***λ***	—	—	0.00091* (0.0007)
***σ* ^2^**	0.8057*** (0.0568)	0.8046*** (0.0571)	0.8038*** (0.0569)
***γ***	0.6080*** (0.0299)	0.6083*** (0.0300)	0.6086*** (0.0299)
$\sigma_{u}^{2}$	0.4899*** (0.0565)	0.4895*** (0.0568)	0.4892*** (0.0566)
$\sigma_{v}^{2}$	0.3158*** (0.0126)	0.3151*** (0.0126)	0.3146*** (0.0125)
**Log-likelihood**	–1585.3151***	-1563.6964***	–1562.7836***
**Wald chi2**	24055.55***	25002.51***	24730.85***

## 5. Conclusions

Taking the listed companies of intelligent manufacturing industry in China as the research sample, combined with the dual lag effect of time and space, the stochastic frontier panel model, spatial lag stochastic frontier model and dynamic spatial lag stochastic frontier panel model are constructed respectively based on four-dimensional factor capitals to measure capital allocation efficiencies of intelligent manufacturing enterprises. We find that all the coefficients of the three models are significant, and the estimated value of the logarithm likelihood function is the best in the dynamic spatial lag panel model. It shows that it is more reasonable to estimate the parameters and measure capital allocation efficiencies of the intelligent manufacturing enterprise by using the dynamic spatial lag stochastic frontier panel model. The development of intelligent manufacturing industry has significant space-time spillover effect among provinces.

Our results show that human capital is the key factor capital of intelligent manufacturing enterprise performance, followed by technological capital and knowledge capital. Therefore, intelligent manufacturing enterprises should pay attention to the investment of human capital, especially the investment of heterogeneous human capital. Human capital is the premise for enterprises to introduce, digest and absorb advanced technology, equipment and management experience as well as transform it into production efficiency. Only when an enterprise has sufficient human capital can it absorb and innovate advanced technology and mature management experience, and increase its knowledge capital and technological capital through the knowledge accumulation and progress.

From the perspective of capital allocation efficiencies, about 52.98% intelligent manufacturing enterprises are at a high level, but 12.04% of them still need to optimize capital allocation. The gap between the actual performance of sample enterprises and the efficiency frontier is mainly due to technical ineffectiveness. An enterprise should further coordinate knowledge capital and technological capital under the existing conditions of material capital and human capital, to realize the balanced allocation of each factor capital, to promote the growth and development of enterprises. From a regional perspective, the top ten enterprises with the highest capital allocation efficiency are all in the eastern region, but the average value of capital allocation efficiency is the highest in the western region, followed by the eastern and central regions. Therefore, the government should actively improve the factor capital trading market, provide a trading platform and mechanism for the optimal allocation of enterprise factor capital, promote the integration and rational allocation of resources, and promote the adjustment of industrial structure.

## Supporting information

S1 Data (XLSX)
